# Reply to “Comment on ‘Glass Transition, Crystallization of Glass-Forming Melts, and Entropy”’ by Zanotto and Mauro

**DOI:** 10.3390/e20090704

**Published:** 2018-09-13

**Authors:** Jürn W. P. Schmelzer, Timur V. Tropin

**Affiliations:** 1 Institut für Physik der Universität Rostock, Albert-Einstein-Strasse 23–25, 18059 Rostock, Germany; 2 Frank Laboratory of Neutron Physics, Joint Institute for Nuclear Research, ul. Joliot-Curie 6, 141980 Dubna, Russia

**Keywords:** glasses, glass transition, crystal nucleation, entropy, 64.60.Bd General theory of phase transitions, 64.60.Q- Nucleation, 64.70.D- Solid-liquid transitions, 64.70.kj Glasses, 64.70.Q- Theory and modeling of the glass transition, 65.40.gd Entropy

## Abstract

A response is given to a comment of Zanotto and Mauro on our paper published in Entropy **20**, 103 (2018). Our arguments presented in this paper are widely ignored by them, and no new considerations are outlined in the comment, which would require a revision of our conclusions. For this reason, we restrict ourselves here to a brief response, supplementing it by some additional arguments in favor of our point of view not included in our above-cited paper.

## 1. Introduction

The main part of our paper [1] and the comment on it [2] are concerned with the questions: (i) whether continuous relaxation has to be included in the definition of glass; (ii) whether glasses always crystallize; finally (as suggested by Zanotto and Mauro in [3]), (iii) how kinetic criteria of glass transition can be formulated most appropriately; and (iv) whether glasses have a residual entropy or not. The differences between our and Zanotto and Mauro’s points of view were described comprehensively in our paper [1]. Therefore, we provide here a brief response and supplement it by additional arguments not included in [1].

## 2. Main Topics of Controversy

### 2.1. Definition of Glass and the Glass Transition

**Figure 1:**
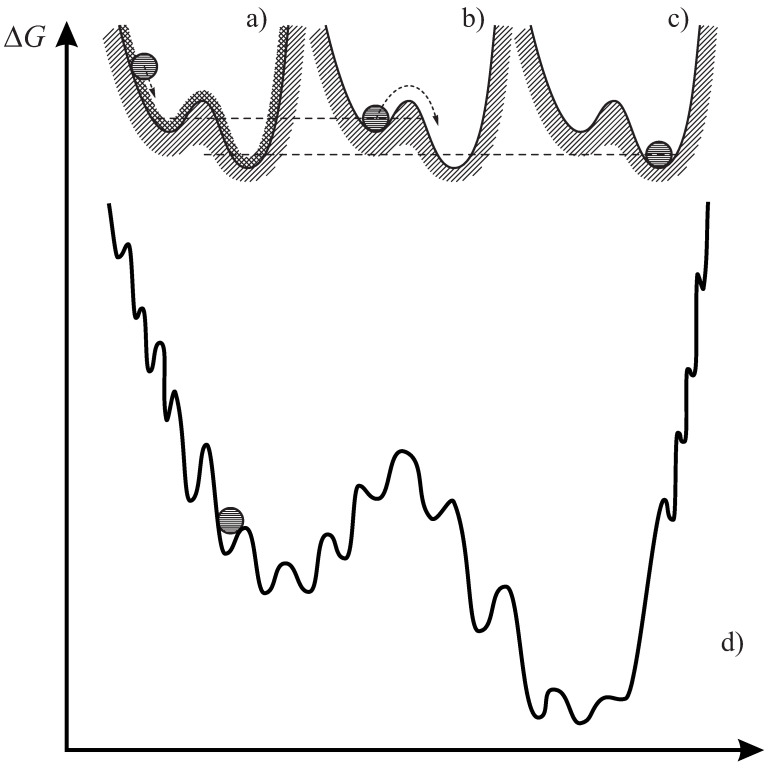
Mechanical analogy for an interpretation of the differences between (**a**) the glass, (**b**) the metastable liquid and (**c**) the stable at $T<T_{m}$ crystalline state ($T_{m}$ is the melting or liquidus temperature). In this mechanical analogy, the crystalline state corresponds to an absolute minimum of the (thermodynamic) potential well, the under-cooled melt to a higher local minimum. In order to be transferred from the metastable to the stable crystalline state, the system has to overcome a potential barrier denoted in nucleation theory as the work of critical cluster formation. The current state of the glass is represented in this analogy by a ball glued to the wall of the potential well above the minimum (**a**). Crystallization, if it occurs, is frequently preceded by stabilization processes, i.e., the approach to the metastable equilibrium state of the liquid [4,5,6]. This is commonly taken as granted in the analysis of crystal nucleation in terms of classical nucleation theory [4,7]. The modifications one has to introduce if this is not the case are described in detail in our papers [8,9]. In (**d**), a modification of Simon’s picture of the vitreous state is given accounting for the potential energy landscape picture of the evolution of glass-forming systems as advanced by Goldstein [10] (see the text).

A minor part of our paper [1] was devoted to different definitions of the glass and the glass transition and the formulation of kinetic criteria determining it as the basis for the subsequent analysis. In this connection, it is worth reminding about the interpretation of the vitreous state and its relation to the metastable liquid, respectively, the crystal phase as developed by Simon. It is reproduced in Figure 1a–c adapted from the monograph by Gutzow and Schmelzer ([4], Figure 2.32). In brief, as formulated first by Simon, glasses are frozen-in non-equilibrium states (for more details, see the caption to Figure 1 and, e.g., [1,4]). The relaxation of a glass to the metastable equilibrium state and its further transformation to a crystal was supposed by Simon to be prevented, as a rule, for any reasonable time scales by kinetic reasons.

Zanotto and Mauro [3] claim that their “new modern ideas” consist of the statement that glasses always relax and finally crystallize. From a thermodynamic point of view, they do not go beyond Simon’s model and the particular way of formation of glasses he was analyzing. New developments in glass sciences since the times of Simon are not reflected in the definition proposed by them. Indeed, Zanotto and Mauro [3] even pose the question whether Simon had already a similar point of view as theirs. In our paper [1], we reproduced in translation a respective statement by Simon showing that this is not the case. As noted by Davies and Jones [11]: “Simon pointed out that as a glass is cooled through its transformation temperature the molecular diffusion which is necessary to effect the appropriate change in configuration is increasingly inhibited and finally becomes practically impossible”.

The existence of long-time flow was known already since the 1850s and even earlier, as can be traced, for example, in the work of Kohlrausch reviewed in [12]. Nemilov and Johari [13] noted that James Prescott Joule had drawn the attention to such flow processes by measuring the zero degree Celsius point over a period of 38.5 years (from April 1844–December 1882). Numerous studies of the change in the density and refractive index of optical glass with time have been performed and published in the years from the early 1930s. Anyway, for most (not all) practical applications, flow and relaxation of glass are taken as irrelevant, and glass is treated as a solid.

Zanotto and Mauro claimed that new developments in glass science require a new or modern definition of glass. However, really new developments are not accounted for in the definition proposed by Zanotto and Mauro, and several statements are simply incorrect, as discussed in [1]. In addition, one could try also really to advance Simon’s picture, supplementing it by potential energy landscape ideas originally proposed by Martin Goldstein [10] (see Figure 1d) and their implementation accounting more appropriately for a combination of the general trends in the possible evolution of glasses formed via glass transition in cooling with details of the evolution of glass-forming melts, respectively glasses. As it seems to us, by such an approach, a variety of details (see, e.g., [14,15,16,17,18]) could be possibly given an interpretation not reflected in the original form of Simon’s model. In such a more general approach, thermodynamic properties of deeply supercooled liquids are dominated by the local potential energy minima, while the kinetics of relaxation and transport is governed by transitions between the local minima as described in a review by Ediger and Harrowell [18].

### 2.2. Greek Philosophy and Kinetic Criteria of Glass Formation

Reiner [19] introduced the Deborah number relying on Heraclitus statement that “Everything flows”. His statement is cited in our paper [1] first to show that (i) since everything flows in historical time scales, it makes no sense to include such a feature into the definition of some particular state of matter. Moreover, (ii) we demonstrated that it is not the relation between experimental observation time, not specified by Zanotto and Mauro in [3], and structural relaxation time that leads to a glass formation in cooling or similar processes, but the interplay between the characteristic time of change of external control parameters (clearly defined by us via their rate of change and, for cooling and heating, the glass transition temperature) and relaxation time. As shown, all specific kinetic criteria proposed in the literature of glass-formation are special (approximate) expressions of the general criterion derived by us [1,4,20]. The Deborah number is introduced by Reiner to distinguish between liquids and solids and not liquids and glasses. It can be adapted to the glass transition, but this has to be done in a correct way as described by us [4,20].

### 2.3. Flow vs. Relaxation

In our paper [1], it is demonstrated that flow and relaxation are interrelated. This correlation is expressed by the Maxwell relation [4] connecting the relaxation time with Newtonian viscosity. Zanotto and Gupta [21,22] used this relation to describe the change in the shape of window glass with time by gravitational flow. Consequently, any attempts to artificially distinguish both processes as independent are incorrect.

Zanotto and Mauro [3] further mention the necessity for introducing a spectrum of relaxation times for describing the properties of glass-forming melts. This necessity is described by us in [1,4]. For the description of relaxation, we employ a relation of the form:

(1) $$\frac{d\xi }{dt}=-\frac{1}{\tau_{R}\left(p,T,\xi \right)}\left(\xi -\xi_{e}\right)\;.$$

here, the relaxation time, $\tau_{R}$, is a function of pressure, *p*, temperature, *T*, and, at least, one structural order-parameter, ξ. We showed in [8,9] that this dependence of the relaxation time on the structural order-parameters may give the key to the understanding of deviations from Maxwell’s relaxation law like the stretched exponential relation. Hence, a solution of a long-standing problem [23] we proposed was how stretched-exponential relaxation can be understood from a theoretical point of view. We also discussed in detail why different quantities relax by different laws and that the dependence of the relaxation time on the structural order-parameter automatically yields a spectrum of relaxation times [24].

### 2.4. Temperature Dependence of the Viscosity

Whether the viscosity diverges at low temperatures (as implied by the Vogel–Fulcher–Tammann (VFT) equation [4]) or not is a matter of debate [25,26,27]. This problem cannot be resolved by direct experimental investigations restricted to maximum values of viscosity $\eta <10^{18}$ Pa·s. In case the predictions of VFT or similar relations hold true, the definition of glass proposed by Zanotto and Mauro is invalid not only for practical purposes, but also from a principal point of view.

The advantages of the VFT-equation have been noted also in [28] by one of the authors of the Comment [3], claiming to have given there a statistical-mechanical derivation of another empirical model established experimentally by Waterton in 1932 [29]. As noted in [4], even earlier, this relation was proposed by le Chatelier. It was then widely employed by Schischakov for describing the temperature dependence of the viscosity. To denote the le Chatelier-Waterton-Schischakov equation as the MYEGA-equation we consider consequently as misleading.

Having stressed in [28] the absence of a divergence of the viscosity at low temperatures as one of the advantages of the le Chatelier–Waterton–Schischakov equation, Mauro joins some years later a group of authors [30] stating the opposite: a divergence of viscosity and/or relaxation time does occur, and the temperatures of divergence of the relaxation time and the Kauzmann temperature (stated in contrast to [28] to exist in accordance also with a variety of other investigations (see [1,31,32])) coincide. At least for these 55 liquids, respectively, glasses analyzed in [30], there exist ranges of temperature and pressure, where (as noted above) relaxation and crystallization are principally excluded.

Finally, in our discussions in [1], we focused attention on qualitative features and mentioned that the conclusions derived by us do not depend on any particular choice of the equation for describing the viscosity. That the viscosity does, in general, depend also on the degree of deviation from equilibrium is well known [4,9], but it is irrelevant for the purposes under consideration here.

### 2.5. Crystallization

That glasses may crystallize is not a matter of discussion; the question is whether glasses always finally crystallize or not. Several examples are provided in our paper [1] showing that this is not the case. This conclusion is confirmed by a recent computer simulation of crystallization and glass transition [33] and also by the “paradox of old glasses” as formulated by Berthier and Ediger [34] (glasses do not crystallize at normal conditions in relevant time scales). Moreover, some of the most frequently-used polymer glasses, namely atactic poly(methyl methacrylate), do not crystallize at all. For example, in a recent paper [35] entitled “The Ultimate Fate of Supercooled Liquids” Stephenson and Wolynes concluded that “some atactic polymers or heteropolymers may not be able to crystallize at all because they have no plausible competing periodic crystal structure, most everyday glass substances are only kinetically prevented from crystallizing on human time scales”.

In [4,36], Tammann’s development method is discussed as a major tool in experimental analysis of crystallization. It had been developed by Tammann long ago and is widely employed in the analysis of crystal nucleation in glass-forming melts. The reason is that at the temperatures where crystals may nucleate, the nuclei frequently do not grow. Moreover, also Zanotto et al. have drawn attention to the fact that “very few silicate glasses show internal homogeneous nucleation” [37].

### 2.6. Broken Ergodicity and Entropy

In [1], we concluded that glasses do have a residual entropy in agreement with the well-established point of view as advanced in the previous century. There is no need to wait for “the ultimate truth (that) must come from experiments” (as stated in [2]). Such a suggestion was already formulated about a decade ago [38]. A variety of such experiments do exist, and they are described in [1] and in the references cited therein supporting the traditional point of view. Previously claimed experimental proofs of their alternative point of view, like the one advanced in [39], are shown to be incorrect in [40]. We further illustrated our conclusions by a simple model based on statistical mechanical models and thermodynamics of irreversible processes. All essential features of the glass transition are reproduced by accounting for the increase of viscosity and/or relaxation time with decreasing temperature.

In [1], we already discussed the paper by Goldstein [41] showing that a zero value of the residual entropy violates the second law of thermodynamics. However, even if such a consequence is accepted, the approach followed by Mauro et al. leads to internal inconsistencies as elaborated in detail by P. Gujrati [42]. In addition, a comprehensive analysis of theoretical aspects of the problems under consideration has been performed by Nemilov [43]) resulting in the conclusion: “If we rely upon the classical works of Gibbs, Planck, Einstein, Fermi, Prigogine, and other authors of modern physics, it is impossible to accept the limitations of the thermodynamic consideration of the vitreous state proposed by Gupta, Mauro and co-authors”.

## 3. Final Remarks

Summarizing, the main part of our paper [1] and the comment on it [2] are concerned with the questions whether (i) the aspect of continuous relaxation has to be included into the definition of glass and (ii) whether glasses always crystallize, ultimately. We continue to follow the point of view (in line with the fathers of glass science (like Tammann, Simon, frequently referred to here and by many others)) that—since everything flows at large time scales—the first point is not a distinguishing feature that has to be included into the definition of a particular state of matter. Examples are given that for some glasses, relaxation and crystallization are completely excluded, so both are not general features that need to be included in the definition of glass. Finally, (iii) general kinetic criteria of glass transition can be formulated relying on the relation between characteristic times of change of external control parameters and relaxation time, and (iv) glasses do have a residual entropy, as established theoretically and experimentally by numerous outstanding scientists long ago.
